# Food Additive Sodium Benzoate (NaB) Activates NFκB and Induces Apoptosis in HCT116 Cells

**DOI:** 10.3390/molecules23040723

**Published:** 2018-03-22

**Authors:** Betul Yilmaz, Arzu Zeynep Karabay

**Affiliations:** Faculty of Pharmacy, Department of Biochemistry, Ankara University, 06100 Ankara, Turkey; betulay@gmail.com

**Keywords:** NaB, colon, cancer, apoptosis, NFκB

## Abstract

NaB, the metabolite of cinnamon and sodium salt of benzoic acid is a commonly used food and beverage preservative. Various studies have investigated NaB for its effects on different cellular models. However, the effects of NaB on cancer cell viability signaling is substantially unknown. In this study, the effects of NaB on viability parameters and NFκB, one of the most important regulators in apoptosis, were examined in HCT116 colon cancer cells. Cell culture, light microscopy, spectrophotometry, flow cytometry, and western blot were used as methods to determine cell viability, caspase-3 activity, NFκB, Bcl-xl, Bim, and PARP proteins, respectively. NaB (6.25 mM–50 mM) treatment inhibited cell viability by inducing apoptosis, which was evident with increased Annexin V-PE staining and caspase-3 activity. NFκB activation accompanied the induction of apoptosis in NaB treated cells. Inhibition of NFκB with BAY 11-7082 did not show a pronounced effect on cell viability but induced a more apoptotic profile, which was confirmed by increased PARP fragmentation and caspase-3 activity. This effect was mostly evident at 50 mM concentration of NaB. Bcl-xl levels were not affected by NaB or BAY 11-7082/NaB treatment; whereas, total Bim increased with NaB treatment. Inhibition of NFκB activity further increased Bim levels. Overall, these results suggest that NaB induces apoptosis and activates NFκB in HCT116 colon cancer cells. Activation of NFκB emerges as target in an attempt to protect cells against apoptosis.

## 1. Introduction

NaB (sodium benzoate), the metabolite of cinnamon and sodium salt of benzoic acid, is a commonly used food and beverage preservative, classified as a GRAS (Generally Recognized As Safe) compound by the FDA (Food and Drug Administration) [1]. NaB is also used as a treatment for disorders of the urea cycle and hyperammonemia because of its ability to bind and remove nitrogenous residues and decrease excess ammonia levels [2]. Since it is one of the most widely used preservatives worldwide, the biological effects of NaB have been studied in different in vitro and in vivo models. It has been shown that NaB maintains and upregulates regulatory T cells [3,4], induces IL-4 production by human peripheral bone mononuclear cells [5], and suppresses Th1-type immune responses [6]. As part of its immune modulatory function, NaB has been shown to inhibit leptin release in an in vitro murine adipocyte model, which indicates the role of this additive in the development of obesity [7]. Myelinogenic properties and memory protective activities in Multiple Sclerosis and Alzheimer’s disease due to NaB have also been reported [8,9]. Toxicity induced by NaB has also been studied at different levels. NaB exhibits mutagenic and cytotoxic activity in lymphocytes and splenocytes at concentrations of 2.0 mg/mL and 2500 µg/mL, respectively [10,11], whereas induced neurotoxicity, nephrotoxicity, and lethality of zebrafish larvae occurs at 2000 ppm [12] concentration. Taken together, this research data partly reveals different biological effects and modulation of cytotoxicity by NaB. On the other hand, to our knowledge, no research exists addressing the effects of NaB on proliferation status and apoptotic signaling in cancer.

In recent years, the link between dietary factors and different cancers has been extensively studied [13,14,15,16]. Among these cancers, colorectal cancer has been reported as the third most common cancer worldwide, with nearly 1.4 million new cases diagnosed in 2012 according to the World Cancer Report 2014 [17]. Development of colorectal cancer is closely linked to inflammatory pathways and chronic inflammation [18]. One of the key modulators of inflammation and immune function is NFκB transcription factor, which is implicated in the pathogenesis of both hematological and solid cancers, including colon cancer [19]. Multiple dietary compounds have been shown to modulate NFκB activity and play a role either in the prevention or progression of cancer [20]. NaB has been reported to inhibit NFκB activity in microglial cells at its non-cytotoxic concentrations and decrease NFκB gene expression in the liver tissues of mice [21,22]. However, the effects of NaB on NFκB activity and the modulation of cell viability in cancer is not known. In light of these data, in this study we aimed to investigate the effects of the widely used food and beverage preservative NaB on the cell viability of HCT116 colon cancer cells through NFκB modulation.

## 2. Results

### 2.1. NaB Inhibited Proliferation of HCT116 Colon Cancer Cells at Concentrations Higher than 6 mM

**Figure 1:**
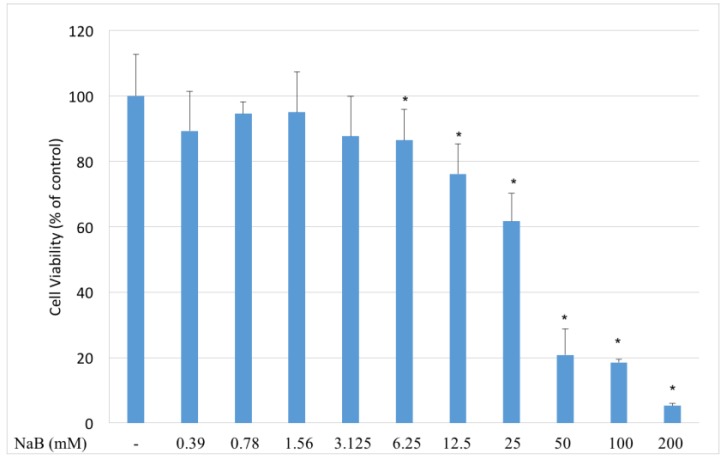
Modulation of HCT116 cell viability by NaB. HCT116 colon cancer cells were seeded to 96 well plates and after one night incubation, they were incubated with 0.39–200 mM concentrations of NaB for 24 h, before detecting cell viability with a MTT test. NaB inhibited cell viability between 6.25–200 mM concentrations significantly (*p* < 0.05). The decrease in cell viability was dose dependent between 6.25–200 mM concentrations, except no significant difference was detected between the cell viability of cells treated with 50 and 100 mM sodium benzoate.

To identify the effects of NaB on cell proliferation, HCT-116 colon cancer cells were incubated with 0.39–200 mM concentrations of NaB before detecting cell viability with a MTT [3-(4,5-Dimethylthiazol-2-yl)-2,5 diphenyltetrazolium bromide] test. Our results showed that NaB inhibited the cell viability of HCT-116 cells significantly (*p* < 0.05) at 6.25 mM and higher concentrations (Figure 1).

### 2.2. NaB Treatment Induced Morphological Changes in HCT116 Colon Cancer Cells

**Figure 2:**
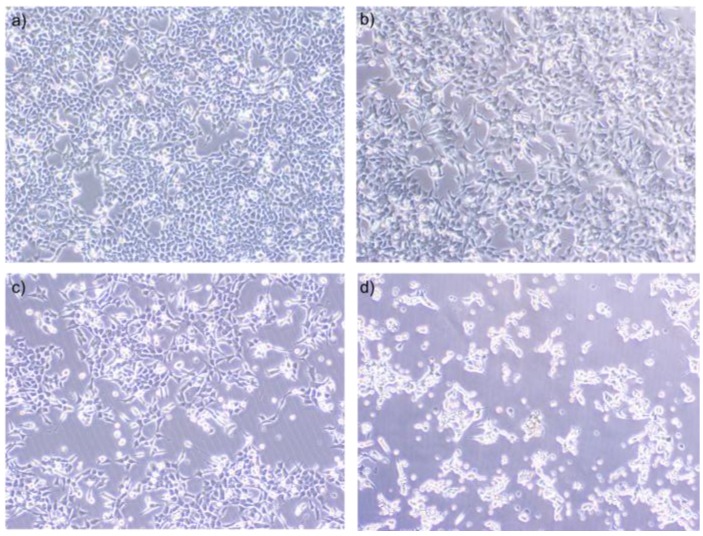
Morphological examination (10× magnification) of NaB treated HCT116 cells under a light microscope (Olympus CKX-53). HCT116 colon cancer cells were seeded to six well plates and the next day they were treated with 6.25–50 mM concentrations of NaB for 24 h. (**a**) Cells treated without NaB; (**b**) Cells treated with 12.5 mM NaB; (**c**) Cells treated with 25 mM NaB; and (**d**) Cells treated with 50 mM NaB. Cells began to lose contact and detach with increasing concentrations of NaB. Healthy morphologic features and cellular integrity completely disappeared and dead cells were clearly seen when cells were treated with 50 mM NaB.

When cells were visualised with light microscopy, it was seen that cells began to lose contact and detach with increasing concentrations (12.5 mM, 25 mM, and 50 mM) of NaB (Figure 2b–d). Healthy morphologic features and cellular integrity (Figure 2a) completely disappeared and dead cells were clearly seen when cells were treated with 50 mM NaB (Figure 2d).

### 2.3. Effect of NaCl on the Viability of HCT116 Colon Cancer Cells

**Figure 3:**
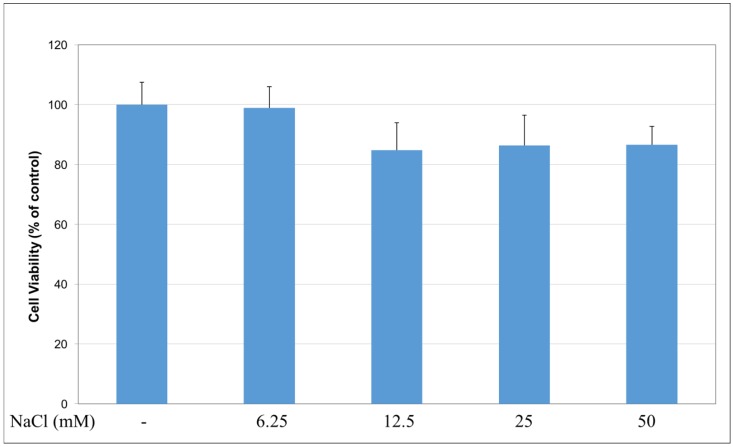
Effect of NaCl on HCT116 cell viability. Cells were treated with 6.25–50 mM concentrations of NaCl for 24 h before detecting cell viability with a MTT test. NaCl treatment (6.25–50 mM) did not show a significant effect on the viability of HCT116 cells.

To reveal if decreased cell viability and altered cell morphology induced by NaB treatment stemmed from an osmotic effect or not, cells were treated with 6.25–50 mM concentrations of NaCl salt as a control, which exhibited the same osmotic pressure with NaB. Our results showed that NaCl treatment did not inhibit cell viability significantly at this concentration range, which suggests that the cytotoxic activity induced by NaB was independent from a possible osmotic effect (Figure 3).

### 2.4. NaB Exhibited Less Cytotoxic Activity on L929 Fibroblast Cells Compared to HCT116 Cells

**Figure 4:**
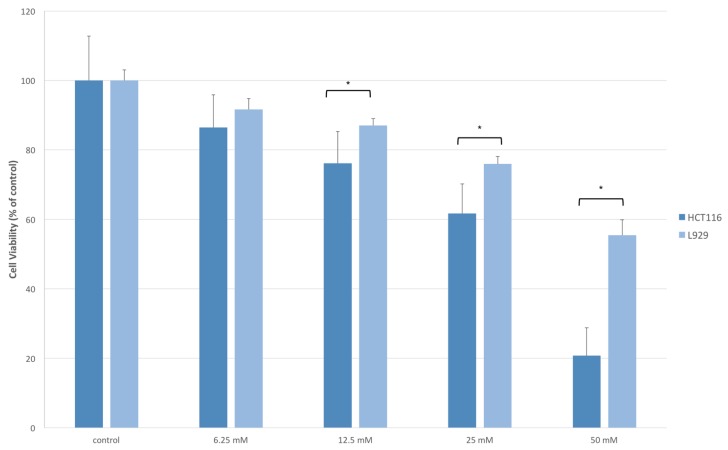
HCT116 colon cancer cells and L929 fibroblast cells were treated with 6.25–50 mM concentrations of NaB before detecting cell viability with a MTT assay. The 6.25 mM NaB treatment did not show a significant effect on the cell viability of L929 cells; whereas, the 12.5–50 mM NaB treatment significantly inhibited L929 cell viability (*p* < 0.05). NaB (12.5–50 mM) treatment exhibited more cytotoxic activity towards HCT116 cells compared to L929 cells (*p* < 0.05).

To test the effects of NaB on the cell viability of a non-tumorigenic cell line, L929 fibroblast cells were treated with 6.25–50 mM concentrations of NaB for 24 h before determining cell viability with a MTT test. Our results showed that 6.25 mM NaB did not have a significant cytotoxic effect on the L929 cell line. On the other hand, 12.5–50 mM concentrations of NaB inhibited cell viability significantly (*p* < 0.05) in L929 cells. When the cytotoxic activity of NaB on HCT116 and L929 cells were compared, it was found that NaB exhibited more cytotoxic activity on HCT116 cells than L929 cells at the same concentrations (Figure 4).

### 2.5. NaB Induced Loss in HCT116 Cell Viability Was Mediated by Apoptosis

**Figure 5:**
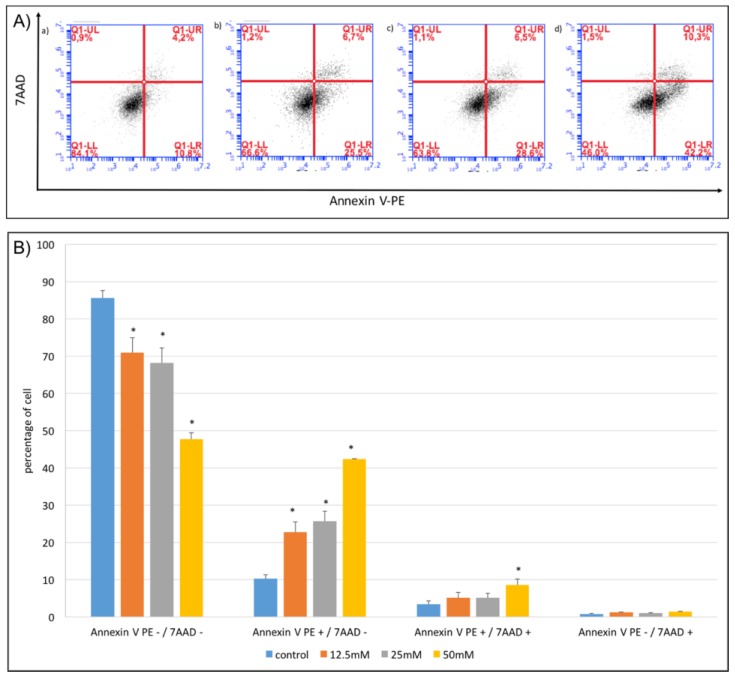
Induction of HCT116 colon cancer cell apoptosis by sodium benzoate. HCT116 colon cancer cells were seeded to six well plates and after overnight incubation, they were treated with 12.5–50 mM concentrations of NaB for 24 h before analysing. (**A**) The representative plots for (**a**) HCT116 cells without treatment, (**b**) HCT116 cells treated with 12.5 mM NaB, (**c**) HCT116 cells treated with 25 mM NaB, and (**d**) HCT116 cells treated with 50 mM NaB. (**B**) Bar graphs for each treatment group stained with Annexin V-PE and 7-AAD before detection with flow cytometry (Accuri C6). PE-/7 AAD- cells represent viable cells, PE+/7 AAD- cells represent early apoptotic cells, PE+/7 AAD+ cells represent late apoptotic cells, and PE-/7 AAD+ cells represent necrotic cells. Our results showed that 12.5–25 mM concentrations of NaB induced early apoptosis (PE+/7-AAD-) (*p* < 0.05); whereas, a 50 mM concentration of NaB induced both early (PE+/7-AAD-) and late (PE+/7-AAD+) apoptosis of HCT116 cells, at a significant level (*p* < 0.05). With a 50 mM NaB treatment, the number of viable cells decreased and induced the number of early apoptotic and late apoptotic HCT116 cells significantly (*p* < 0.05) compared to the 6.25 mM, 12.5 mM, and 25 mM NaB treated groups.

Next, we examined which type of cell death was induced with NaB treatment. After proper treatment and incubation of cells with NaB for 24 h, Annexin V-PE (Annexin V-phycoerythrin)/7-AAD (7-aminoactinomycin D) staining was used to detect apoptosis and necrosis. We found that 12.5–25 mM concentrations of NaB induced early apoptosis (PE+/7-AAD) (Figure 5A,B); whereas, a 50 mM concentration of NaB induced both early apoptosis (PE+/7-AAD) and late apoptosis (PE+/7-AAD+) (Figure 5A,B) of HCT116 cells, at a significant level (*p* < 0.05).

### 2.6. NaB Induced Caspase-3 Activation in HCT116 Cells at Its Apoptotic Concentrations

**Figure 6:**
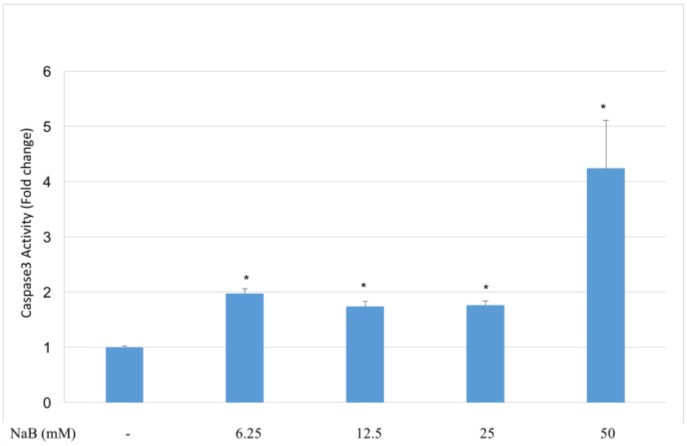
Induction of caspase-3 activity in HCT116 colon cancer cells by sodium benzoate HCT116 cells, incubated with NaB for 24 h. After proper incubation, cells were lysed and cellular extracts containing 100 µg protein were incubated with DEVD-pNA substrate for colorimetric detection. Our results showed that NaB induced caspase-3 activity significantly (*p* < 0.05) at concentrations of 12.5–50 mM compared to untreated cells. The highest caspase-3 activity was achieved by the treatment of cells with 50 mM NaB. This 50 mM NaB treatment increased caspase-3 activity significantly (*p* < 0.05) compared to the 6.25 mM, 12.5 mM and 25 mM NaB treated groups.

HCT116 cells were incubated with NaB for 24 h before measuring caspase-3 activity in the cell lysates. NaB induced caspase-3 activity significantly (*p* < 0.05) at concentrations of 12.5–50 mM compared to untreated cells (Figure 6). The highest caspase-3 activity was achieved by the treatment of cells with 50 mM NaB. Dose dependent activation of caspase-3 was found by 50 mM NaB treatment compared to all other groups.

### 2.7. NFκB Activation Accompanies NaB Induced Loss of Cell Viability and Inhibition of NFκB Activation did not Induce a Massive Effect on Cell Viability, but Induced a More Apoptotic Profile Which May Involve Changed Bim but not Bcl-xl Expression

**Figure 7:**
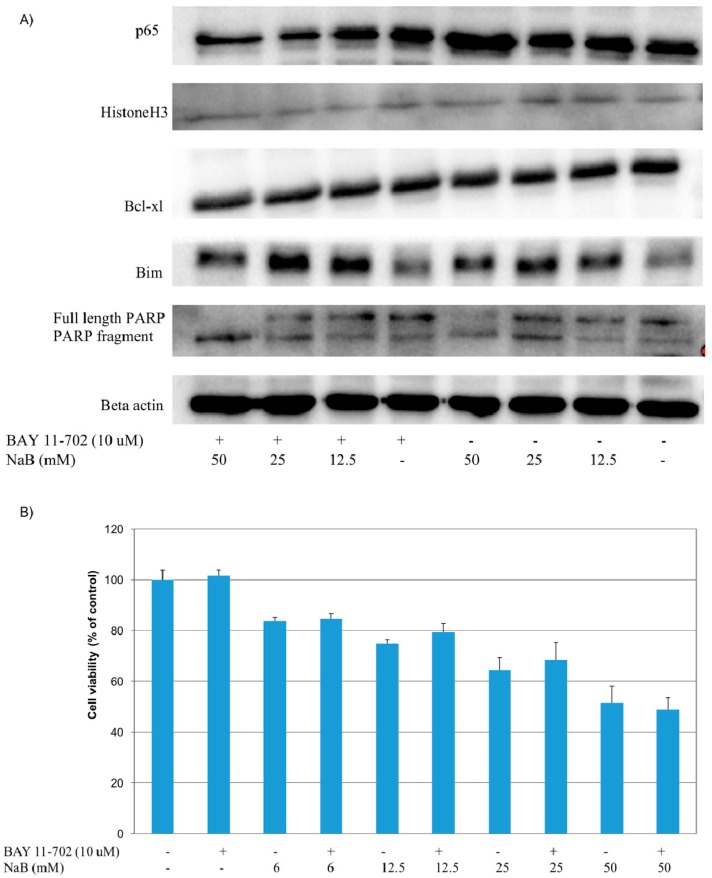

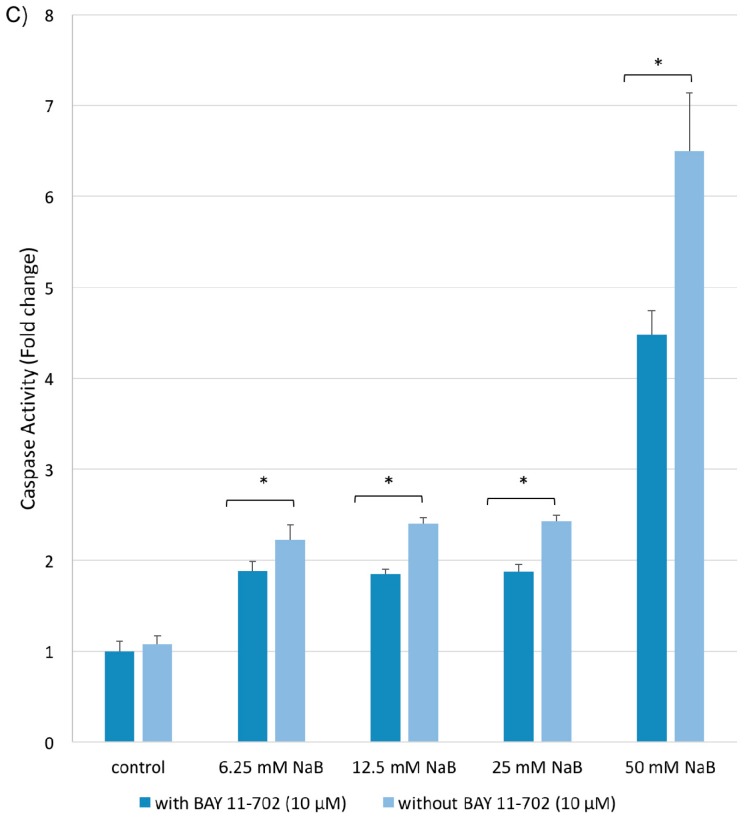
Effects of NaB and NaB/BAY 11-7082 treatment on NFκB activation, cell viability, PARP fragmentation, and Bim and Bcl-xl proteins, in HCT116 colon cancer cells. HCT116 colon cancer cells were seeded to 96 and six well plates and after overnight incubation, cells were treated with or without 10 µM BAY 11-7082 for one hour before incubation with NaB (12.5–50 mM) for 24 h. At the end of the incubation period, cells in the 96 well plates were incubated with a MTT reagent to determine cell viability; whereas, the cells in the six well plates cells were collected and lysed. Nuclear and total cell fractions were blotted for NFκB-p65, PARP, Bim, and Bcl-xl proteins and assayed for caspase-3 activity. (**A**) NaB treatment significantly increased nuclear NFκB-p65 levels and PARP fragmentation in HCT116 cells (*p* < 0.05). The treatment of cells with the NFκB inhibitor BAY 11-7082 resulted in decreased nuclear NFκB -p65 levels. Cells treated with NaB alone exhibited increased Bim expression (*p* < 0.05) compared to controls. When the NFκB inhibitor BAY 11-7082 was applied to cells in combination with NaB, we found that Bim expression was significantly (*p* < 0.05) higher than cells treated with NaB (12.5 and 25 mM) alone. A significant difference in the Bcl-xl levels between NaB treated and untreated cells could not be detected. BAY 11-7082 application also did not affect total Bcl-xl expression in HCT116 colon cancer cells. (**B**) The viability of cells did not exhibit statistical significance between BAY 11-7082 treated and untreated cells at the same concentrations of NaB. (**C**) Caspase-3 activity in BAY 11-7082 pre-treated and untreated HCT116 cells at the same concentrations (6.25–50 mM) of NaB were examined and it was found that caspase-3 activity was significantly higher in cells that were pre-treated with BAY 11-7082.

Since recent reports suggest that NaB affects NFκB activation [21,22], we next tested if nuclear levels of NFκB-p65 changed with NaB treatment. After the incubation of cells with NaB (12–50 mM) for 24 h, cells were collected and the NFκB-p65 protein was detected in the nuclear extracts with western blot. Our results showed that NaB treatment resulted in increased levels of the NFκB-p65 protein (*p* < 0.05) in the nucleus compared to untreated cells (Figure 7A). NaB treatment at a 50 mM concentration caused the highest level of nuclear NFκB-p65 protein, at a significant level (*p* < 0.05) (Figure 7A). These results showed that NFκB-p65 translocation to the nucleus occurs during the NaB treatment of HCT116 cells.

Due to different modulatory roles of NFκB in the modulation of cell death, we inhibited NFκB NFκB-p65 translocation with BAY 11-7082 to reveal the function of NFκB activation in NaB treated cells and tested cell viability. For this purpose, HCT116 colon cancer cells were first incubated with NFκB inhibitor BAY 11-7082 for 1 h, before treatment of cells with NaB for 24 h. To confirm the inhibition of NFκB activity, nuclear NFκB-p65 levels were detected with western blot. It was found that BAY 11-7082 treatment resulted in decreased nuclear NFκB levels (*p* < 0.05) (Figure 7A). We also determined cell viability after NFκB inhibition and our results showed that NFκB inhibition did not exhibit a significant effect on the viability of HCT116 colon cancer cells treated with or without the same concentrations of NaB (Figure 7B). Even if we did not find a significant change in cell viability, we wanted to check the apoptotic status of cells treated with or without NaB. We therefore checked PARP (poly ADP ribose polymerase) fragmentation and found that PARP fragmentation was higher in cells treated with a BAY 11-7082/NaB combination compared to cells treated with NaB alone. Especially at 50 mM concentration, which was the concentration that caused the highest NFκB activation, inhibition of NFκB activity resulted with the disappearance of full length PARP and increased PARP 89 kDa fragments, which are indicators of apoptosis induction (Figure 7A). We thus concluded that NFκB inhibition may have caused a shift to apoptosis in the NaB treated cells.

We next tested if the blockage of NFκB activity caused any effect on the total protein levels of the known NFκB targets, Bim and Bcl-xl. Our results showed that, cells treated with NaB alone exhibited increased Bim expression (*p* < 0.05) compared to controls (Figure 7A). When the NFκB inhibitor BAY 11-7082 was applied to cells in combination with NaB, we found that Bim expression was significantly (*p* < 0.05) higher in 12.5 and 25 mM NaB treated cells than cells treated with NaB alone, which may support the further induction of apoptosis (Figure 7A). On the other hand, we did not find a significant difference in Bcl-xl levels between NaB treated and untreated cells. BAY 11-7082 application also did not affect total Bcl-xl expression in HCT116 colon cancer cells (Figure 7A). Moreover, caspase-3 activity was also determined in BAY 11-7082 pre-treated cells (Figure 7C). It was found that caspase-3 activity in HCT116 colon cancer cells pre-treated with BAY 11-7082 and treated with 6.25 mM, 12.5 mM, 25 mM, and 50 mM concentrations of NaB was significantly higher than cells that were not pre-treated with BAY 11-7082 and treated with the same respective concentrations of NaB. This data further supports the acclaimed protective activity of NFκB against apoptosis in NaB treated cells.

## 3. Discussion

In this study, we tested the cell viability modulatory effects of NaB on HCT116 colon cancer cells and found that NaB significantly diminished the cell viability of colon cancer cells, beginning at 6 mM and higher concentrations. In the literature, cytotoxic effects of NaB have been investigated in different in vitro and in vivo models. Apart from these studies, according to the OECD SIDS initial assessment report on benzoates, for the salts of benzoic acid, repeated dose (long-term inclusive) oral toxicity gives a NOAEL (no observed adverse effect level) of >1000 mg/kg/day [23]. In a study carried out on pregnant wistar rats fed with 700, 1400, 2800, or 5600 mg/kg/day doses of NaB for 20 days gestation, a fetal and maternal NOAEL of 1400 mg/kg/day was determined, based on reduced food intake and decreased body weight in the pregnant rats, perinatal death, organ abnormalities, and skeletal abnormalities, which were found as a result of reduced maternal feed intake and malnutrition [24]. These oral doses have been used and reported for NaB. However, a certain translation of its tissue concentration cannot be made due to differences in metabolism, pharmacokinetics, and bioavailability. When we checked the applied concentrations of NaB in different cell lines we found that NaB has been used in the mM range. NaB was reported to be non-cytotoxic for splenocytes and lympocytes under 1000 µg/mL (7 mM) and 2000 µg/mL (14 mM) concentrations, respectively [5,11]. Maier et al. reported that NaB treatment did not affect cell viability up to 50 mM in concentration [6] in human peripheral blood mononuclear cells; whereas, a 20 mM NaB treatment was reported to have no influence on cell viability in adipocytes [7]. We could not find any study revealing its effects on cancer cells and according to our data, 6.25 mM is the starting concentration of NaB for decreasing cell viability in colon cancer cells. We also checked the cytotoxic effect of NaB on non-tumorigenic L929 fibroblast cells and found that it exhibited significant cytotoxicity at the 12.5–50 mM concentration range. However, NaB induced cytotoxicity was more potent in HCT116 colon cancer cells compared to L929 fibroblasts. The cytotoxicity achieved with different concentrations of NaB in our study, as well as these other studies, suggest cell-specific effects of this additive. 

NaB treatment induced the number of Annexin V-PE stained apoptotic cells and caspase-3 activity significantly at the 12.5–50 mM higher concentration range which showed that the mode of cell death induced by NaB is apoptotic. We next examined which mechanisms may have mediated the apoptotic activity. One of the most important signaling pathways in cancer is the NFκB pathway, which has been reported to transactivate genes associated with inflammation, proliferation, apoptosis, metastasis and invasion, and therefore play an essential role in tumorigenesis [25]. Activation of the NFκB transcription factor occurs by the nuclear translocation of the NFκB-p65 component of the NFκB complex [26]. NaB has been shown to inhibit NFκB activity in mouse BV-2 microglial cells at 0.5–2 mM concentrations [21] and decrease NFκB gene expression in the liver tissues of mice [22]. Contrary to these literature data, which show NFκB inhibitory activity for NaB, we found that NaB increased nuclear NFκB-p65 protein levels and NFκB activation of HCT116 colon cancer cells at its cytotoxic concentrations. Our results of increased NFκB activation by NaB treatment may stem from the cell type or the concentration range of the NaB we used. NFκB activation has been shown to inhibit apoptosis by inducing Bcl-2 family members and caspase inhibitors [27]. Accumulation of the IkBα protein by proteosomal inhibition has also been shown to inhibit NFκB activation and induce apoptosis in tumor cells [28]. Inhibition of NFκB activity has also been proposed as a possible mechanism for apoptosis induced by transfer of the wt-p53 gene in human colon cancer cells [29]. These studies reveal that NFκB activation particularly acts as an anti-apoptotic stimuli and inhibition of NFκB activity may exhibit anti-cancer effects. On the other hand, some drugs have been shown to induce apoptosis by NFκB activation. The apoptotic effects of aspirin on HT29 cells have been reported to be mediated by NFκB activation [30]. NFκB activation has also been shown to increase cell death induced by antimitotic agents [31] and different agents, including ascorbyl stearate and arsenite, have been shown to induce cell death in cervical and 16-HBE cells respectively [32,33]. It has also been reported that the pro- or anti-apoptotic function of NFκB has been determined by the nature of the stimulation [34] and collectively, even if NFκB is best characterized for its protective activity as a response to proapoptotic stimulation, it can also induce programmed cell death in certain circumstances [35]. In light of these literature findings, we asked whether NFκB activation, induced by NaB treatment, acted as an apoptotic or anti-apoptotic inducer. To reveal the function of NFκB activation, we treated cells with NFκB inhibitor BAY 11-7082 before NaB treatment. According to our MTT results, inhibition of NFκB activity did not show a potent significant effect on cell viability. However, when we checked the PARP fragmentation status of cells, we found that cells treated with BAY 11-7082 exhibited a more fragmented PARP and full length PARP completely disappeared in cells treated with a NaB (50 mM) /BAY 11-7082 combination compared to cells treated with NaB alone. This result showed that the main function of the increased NFκB activity seen in NaB treated cells occurred to protect cells against apoptosis. Even if NFκB is activated to protect cells against apoptosis, the overall effect of NaB treatment on HCT116 cells is apoptotic, which made us think that other components of apoptosis signaling may be responsible for apoptosis induction. Since NFκB controls the expression of both antiapoptotic and proapoptotic genes, we also examined the probable changes in Bcl-xl and Bim proteins, which are known targets for NFκB. Our results showed that NaB treatment increased total Bim protein expression. Similarly to our findings, increased Bim expression has been reported in the induction of apoptosis in various cancer cell types including breast cancer [36], lung cancer [37], leukemia [38], as well as colon cancer [39]. We also examined total Bim protein expression after inhibition of NFκB activity and found that, Bim expression increased in NaB (50 mM)/BAY 11-7082 treated cells compared to cells treated with NaB alone. Increased Bim expression after NFκB inhibition also supported increased PARP fragmentation in these cells, which is indicative of increased apoptosis. On the other hand, we detected slightly decreased Bcl-xl protein expression with NaB treatment, but this was not found to be statistically significant. BAY 11-7082 treatment also did not affect the Bcl-xl level of HCT116 colon cancer cells. In summary, our results showed that NaB inhibited HCT-116 colon cancer cell viability by inducing apoptosis. NFκB activation accompanied the induction of apoptosis but it seemed to be activated as a protector against apoptosis and NFκB activity may have influenced Bim expression. However, considering the complex nature of NFκB, it is hard to suggest that NaB induced NFκB activation mediates its effects by orchestrating only Bim expression. In conclusion, these results contribute to the understanding of the modulation of cancer cell viability by NaB and further research is needed to elicit more components of apoptosis signaling induced by NaB, which is one of the most widely used food additives worldwide.

## 4. Materials and Methods

### 4.1. Cell Line and Chemicals

HCT116 colon cancer cell line was a gift from Bert Vogelstein (Johns Hopkins University); BSA, glycine, PBS, thiazole blue tetrazolium bromide (MTT), and DMSO was purchased from Sigma, Annexin V-PE; 7-AAD was from BD Biosciences; RPMI 1640 medium, fetal bovine serum, penicillin/streptomycin, and L-glutamine was from PAA; the Caspase-3 colorimetric assay kit was from Abcam; the Bradford reagent was from Bio-Rad; primary antibodies for NFκB-p65, PARP, beta actin, Bcl-xl, and Bim were from CST; the histone H3 antibody was from Biolegend; and secondary anti-mouse and anti-rabbit antibodies were from CST.

### 4.2. Cell Culture

HCT116 (HCT116 p53+/+) colon cancer cells and L929 cells were used in this study. Cells were grown in a RPMI 1640 medium containing 2 mmol/L L-glutamine, 100 U/mL penicillin, 100 µg/mL streptomycin, and inactive fetal bovine serum (10%), and incubated at 5% CO_2_ in a saturated incubator, at 37 °C. Trypsinization was used for passaging cells, which reached a confluence of 80%.

### 4.3. Cell Viability Experiments

Different concentrations of NaB stock solutions were prepared in the cell culture medium. HCT116 colon cancer cells were seeded to 96-well plates for overnight incubation. The following day, the supernatant on cells was discarded and NaB stock solutions were added to the wells to achieve 0.39 mM, 0.78 mM, 1.56 mM, 3.125 mM, 6.25 mM, 12.5 mM, 25 mM, 50 mM, and 100 mM concentrations in the wells. Cells without NaB treatment served as the control group. To detect cell viability, MTT solution was prepared in PBS at a concentration of 5 mg/mL [40]. After 24 h of incubation with NaB, the medium of the cells was removed and 20 μL of MTT solution and 100 μL of medium were added to each well and incubated at 37 °C in a CO_2_ incubator. At the end of the incubation, the medium-MTT mixture on the cells was discarded and 100 μL DMSO was added to each well to dissolve the formed formazan crystals. Absorbance was then measured at 550 nm on a spectrophotometer. The absorbance value of cells not treated with NaB was considered 100% viability and viability in other cell groups was calculated as a percentage from this value. To reveal the effects of NaCl as a control for the detection of a possible osmotic effect by NaB, cells were also treated with a 6.25–50 mM concentration range of NaCl. For the treatment of cells with BAY 11-7082, stocks of BAY 11-7082 were prepared in DMSO and the final cellular concentration of BAY 11-7082 was 10 µM. Cells without BAY 11-7082 treatment were also incubated with the same concentration of DMSO.

### 4.4. Caspase-3 Activity Assay

Cell lysates were prepared by lysing HCT116 colon cancer cells that had been treated with NaB at different doses and protein determination in the extracts was made according to the Bradford method. The absorbance of the resulting colorimetric product was measured at a wavelength of 405 nm after lysates containing 100 μg protein were incubated at 37 °C with DEVD-pNA, which was the substrate of the caspase-3 enzyme. Caspase-3 activity in the cells treated with NaB was calculated as fold change relative to the activity of cells without NaB treatment. Caspase-3 activity was also detected in NaB treated cells pre-treated with or without BAY 11-7082.

### 4.5. Flow Cytometry for Detection of Apoptosis by Annexin V-PE and 7-AAD Staining

Cells were seeded to six well plates and incubated with different concentrations of NaB for 24 h. Afterwards, cells were washed twice with cold PBS and then mixed in 1x binding buffer to give a concentration of 10^6^ cells per mL. Five μL of Annexin V-PE and 5 μL of 7-AAD were added to 100 μL of this solution. Cells were then vortexed very lightly and incubated for 15 minutes at room temperature. Following incubation, 400 μL of 1x binding buffer was added onto the cells and flow cytometry was performed within 1 h. Annexin V-PE negative/7-AAD negative (live) cell, Annexin V-PE positive and 7-AAD negative (apoptotic) cell and Annexin V-PE positive and 7-AAD negative (necrotic) cell populations were determined and analysed [41].

### 4.6. Western Blot for NFκB-p65, Bcl-xl, Bim and PARP

To determine NFκB-p65, Bcl-xl, Bim, and PARP protein levels, HCT116 cells were first lysed by following the instructions of the 101 Bio Nuclear Extraction and Active Motive total cell lysis kit manuals. Protein concentrations of nuclear and total cell lysates were determined by Bradford assay and equal ug of proteins from HCT116 cell lysates were loaded on to the wells of SDS polyacrylamide gels. After electrophoresis, proteins were transferred to PVDF (polyvinylidene fluoride) membranes by application of a 100 V voltage for 1 h after removal of the SDS-polyacrylamide gel [42]. Then, membranes were incubated with anti-NFκB-p65, Bcl-xl or Bim primary antibodies overnight at 4 °C. The next step was incubation with horseradish peroxidase (HRP) conjugated secondary antibody for 1 h at room temperature. Following this last incubation, proteins were visualized with chemiluminescence (ECL) agent and density of bands were analysed.

### 4.7. Statistical Analysis

Statistical analysis was performed with the use of Statistixl software (Nedlands, Western Australia, 6009). All data expressed as means and SD were representative of at least three independent experiments. One-way analysis of variance (ANOVA) with Student–Newman–Keul’s post-hoc test was used to evaluate the significances between the examined groups. Values of *p* < 0.05 were considered as statistically significant.
